# Historical and contemporary butterfly genomes reveal genetic erosion in response to land-use intensification

**DOI:** 10.1016/j.isci.2026.116884

**Published:** 2026-07-22

**Authors:** Nathalie Matthey-de-l’Endroit, Daniel Berner

**Affiliations:** 1 Department of Environmental Sciences, Zoology & Evolution, University of Basel, Vesalgasse 1, 4051 Basel, Switzerland

**Keywords:** agriculture, biodiversity, conservation genetics, genetic variation, inbreeding, insect decline, lepidoptera, *Melanargia galathea*, population genomics, population size

## Abstract

Biodiversity loss is typically assessed at the level of species extinctions, yet many populations are declining without the species going extinct. A major global driver of such declines is the intensification of agriculture beginning in the mid-20th century. To assess the genomic consequences of this shift, we compare genome-wide sequence data from historical (1875–1955) and contemporary individuals of a once-ubiquitous European grassland butterfly. Demographic inference reveals population growth over millennia, likely associated with grassland expansion under low-intensity agriculture, followed by a population decline exceeding 99% that peaks during mid-20th century land-use intensification. This rapid population decline is accompanied by the loss of genetic diversity, increased population differentiation, and inbreeding. Overall, our results demonstrate the dual role of agricultural land use in shaping biodiversity: while low-intensity practices historically facilitated population expansion, modern agricultural intensification has driven dramatic population decline and genetic erosion, a trajectory likely shared by many grassland species worldwide.

## Highlights

• Population growth over millennia under traditional, low-intensity land use
• Drastic population decline during mid-20th century agricultural intensification
• Population decline leading to genetic erosion
• Similar genetic erosion despite differences in current land-use intensity

## Graphical abstract

**Figure:**
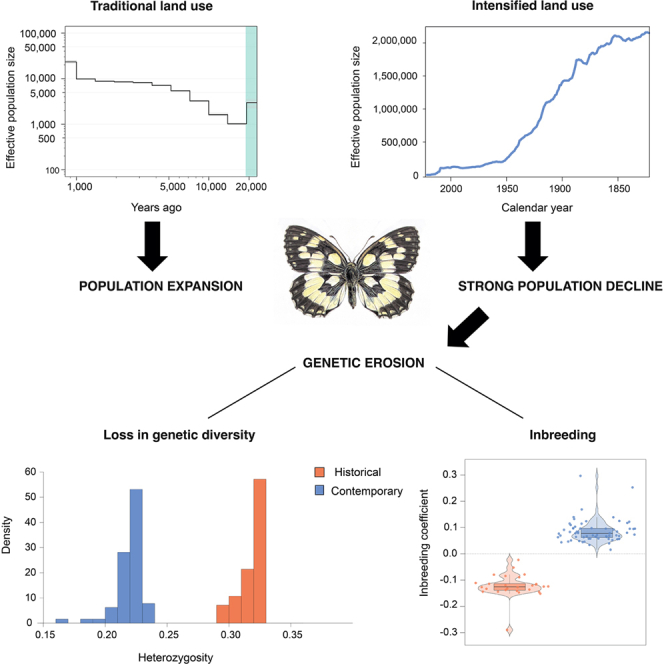

## Teaser

Genomics; Evolutionary biology

## Introduction

The rapid decline of biodiversity is a pressing environmental challenge to humanity, with species losses leading to reduced ecosystem stability and the degradation of benefits that ecosystems provide to humans.^1^^,^^2^^,^^3^^,^^4^^,^^5^^,^^6^^,^^7^ Yet focusing only on species losses greatly underestimates the biodiversity crisis; declines in abundance and local extirpations of populations occur far more frequently and are the precursors to species extinction.^5^^,^^8^

One of the main drivers of worldwide population declines is land-use intensification.^9^^,^^10^^,^^11^ In many regions including Europe, the transition from traditional low-intensity farming to high-input, intensive agriculture peaked between 1940 and 1980 and involved the widespread introduction of synthetic fertilizers, mechanization, and pesticides.^12^^,^^13^^,^^14^^,^^15^^,^^16^ An ecosystem particularly strongly affected by such land-use intensification is semi-natural grasslands that developed over many centuries under traditional low-intensity management practices such as grazing or mowing.^17^^,^^18^^,^^19^^,^^20^^,^^21^ Traditional grasslands hold the world record for fine-scale vascular plant diversity^22^ and in temperate regions represent the most species-rich habitats.^23^^,^^24^

The application of synthetic fertilizers in such biodiversity hotspots shifts plant communities toward the dominance of a few particularly competitive species,^25^ increases standing plant biomass, and hence shifts microclimatic conditions from open, dry, and warm to dense, humid, and cool.^26^^,^^27^ The reduction in plant diversity and the change in microclimate, combined with mechanical disturbance, have cascading negative effects on the associated animal communities.^28^^,^^29^^,^^30^^,^^31^ Accordingly, organisms associated with traditional grasslands have experienced drastic declines since the second half of the 20th century.^16^^,^^32^^,^^33^^,^^34^^,^^35^^,^^36^ Studies documenting these declines, however, have generally focused on abundance or community-level trends,^37^^,^^38^^,^^39^ with comparatively little attention paid to genetic changes within populations.

Population declines intensify genetic drift by reducing effective population size, thereby causing the stochastic loss of potentially beneficial genetic variants, and increasing the probability that deleterious variants rise in frequency and become expressed.^40^^,^^41^^,^^42^^,^^43^^,^^44^^,^^45^ As populations become very small, these elements of genetic erosion are further exacerbated by inbreeding (i.e., mating among close relatives). Limited gene flow among fragmented populations further constrains the replenishment of lost variation. Together, these processes increase extinction risk and reduce populations’ potential to adapt to environmental change.^46^^,^^47^^,^^48^^,^^49^ Beyond these challenges to population persistence, genetic erosion may impair ecosystem function and resilience by constraining trait variation, thereby reducing the breadth of ecological roles that populations can fulfill.^50^^,^^51^^,^^52^

The massive changes in land use during the mid-20th century raise the question of how strongly populations of grassland-associated organisms have declined, and to what extent this had led to genetic erosion. Addressing these questions has been challenging, because scientific and environmental concern about land-use intensification emerged only decades after the major shifts in agricultural practices.^53^^,^^54^ As a result, direct quantitative comparisons of population size and genetic diversity of grassland species before and after major land-use intensification are scarce.^55^

Here, we address this gap using the Marbled White (*Melanargia galathea*), a Western Palearctic butterfly species characteristic of traditional grassland habitats. Historical naturalist accounts from Central Europe consistently describe the Marbeld White as ubiquitously distributed and occurring at high densities in traditionally and extensively managed meadows and pastures.^56^^,^^57^^,^^58^^,^^59^^,^^60^ These habitat types remained widespread until the early 20^th^ century.^20^ After the Second World War, however, agricultural practices in Central Europe intensified dramatically, including the mechanization of farming, the application of synthetic fertilizers and pesticides, and the consolidation of small family farms into larger operations.^13^^,^^15^^,^^16^^,^^32^ As a result, the Marbled White’s occurrence in today’s intensively managed landscapes is restricted to isolated remnant populations.^61^^,^^62^^,^^63^^,^^64^ This grassland butterfly thus serves as a strong system for investigating genetic changes associated with land-use intensification, which we here do based on genome-wide DNA sequence data from historical museum specimens and contemporary samples.

## Results and discussion

### Population rise and decline in response to land use

**Figure 1:**
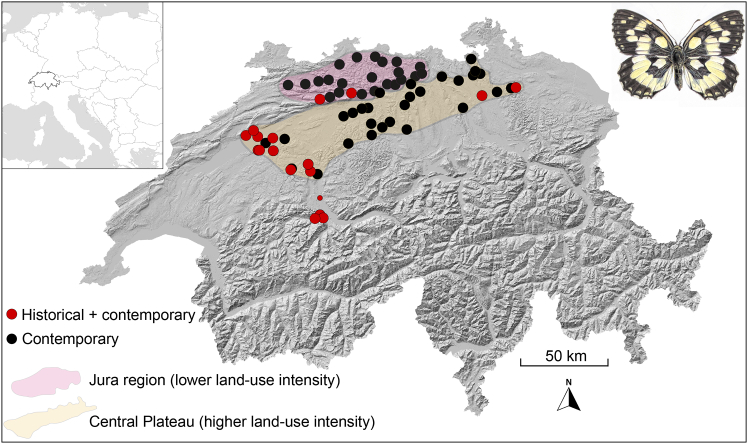
Geographic situation of the study region and sampling localities in Switzerland Red dots indicate localities for which both historical and contemporary butterfly specimens were available and were used for genetic comparisons before versus after agricultural intensification. (The small red dot marks a locality from which only a historical specimen, without a contemporary counterpart, was available.) Black dots denote sites from which only contemporary individuals were sampled. Purple shading highlights the Jura region, characterized by relatively low present-day land-use intensity, whereas yellow shading indicates the Central Plateau dominated by more intensive agriculture. The inset map shows the location of Switzerland within Europe, and the butterfly illustration depicts a female Marbled White (painting by H.-P. Wymann).

To generate the sequence data for our investigation, we used 28 pinned individuals from 17 localities in Switzerland (Figure 1; Table S1) from the collection of the Natural History Museum Bern, Switzerland, spanning from 1875 to 1955 (median collection year: 1929). These museum individuals, collected before major land-use intensification and hereafter referred to as “historical” samples, were whole-genome sequenced. From the same localities, we sampled up to four “contemporary” individuals (64 in total) during the summer of 2023 (see Figure S1 for aerial photographs taken before and after land-use intensification at three exemplary localities). These latter individuals were subjected to reduced-representation (RAD) sequencing. All localities represented by both historical and contemporary individuals are hereafter referred to as “matched localities”.

**Figure 2:**
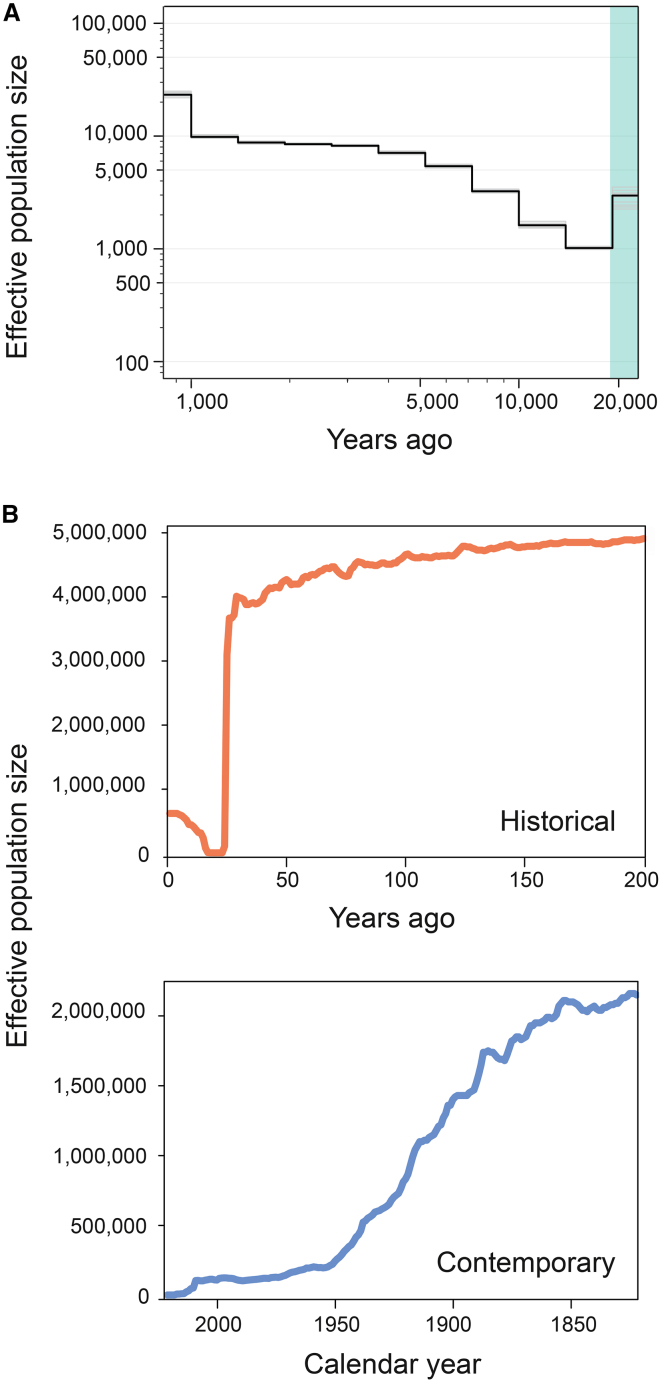
Demographic history of the Marbled White (A) Ancient effective population size through time inferred from whole-genome data from the historical butterfly sample. The black line shows median population size across ten jackknife resamples, and the gray lines display the estimation variation across these replicates. The area shaded light blue marks the last glacial maximum (∼23–19 kya). (B) Recent effective population size inferred up to 200 years (and generations) into the past for the historical and the contemporary samples from the matched localities. The trajectories represent means across 40 independent estimation runs. Because historical specimens were collected over an 80-year interval (1875–1955), the time axis cannot be associated with specific calendar years, contrary to the contemporary sample collected in 2023. For the latter, population size in the time period closest to the present is estimated at approximately 2,500. Note that different analytical approaches were used for panels A and B, hence ancient and recent population size estimates are not directly comparable.

Genetic marker data offer the opportunity to estimate temporal changes in population size. Our first analytical step thus involved identifying single-nucleotide polymorphisms (SNPs) from the historical samples, and from these estimating the “ancient” effective population size of the Marbled White from the end of the last glaciation to approximately thousand years before the present.^65^ The motivation was to assess demographic responses to the spread of traditional, low-intensity land use. This analysis revealed a minimum in population size after the last glacial maximum, followed by rapid recovery over the subsequent millennia, including a pronounced population surge roughly 1,000 years ago (Figure 2A). These demographic trends are consistent with substantial habitat loss and persistence in refugia during the last ice age, followed by population expansion during the subsequent Holocene warming.^66^^,^^67^^,^^68^ Interestingly, the strong population increase toward the present likely reflects rapidly expanding grassland availability due to widespread deforestation,^24^^,^^69^^,^^70^^,^^71^^,^^72^ paralleling increases in human population size across Europe.^73^^,^^74^^,^^75^

Having obtained indication of a strong benefit of human agricultural activity on a grassland species like the Marbled White, we next used our marker data to examine more fine-grained population trends over approximately the last three centuries. Specifically, we estimated effective population size 200 generations backward in time^76^ separately for the historical and for the contemporary individuals from the matched localities. The historical data indicated a largely constant population size in the millions prior to agricultural intensification, followed by a pronounced decline toward the end of the inferred time period (Figure 2B top). Because historical samples were pooled across an ∼80-year interval, the precise timing of the onset of this decline could not be resolved. This was possible with the contemporary data, however, indicating initial population sizes in the millions that began to decline around 1850, dropping to a few thousand in the most recent years—corresponding to a reduction of more than 800-fold (Figure 2B bottom). The most rapid contraction occurred between 1920 and 1960, coinciding with widespread land-use intensification in Central Europe, with further reductions continuing to the present. The plausibility of this extremely small current population size was supported by independent simulations (Figure S2).

### Genetic consequences of the mid-20th century population decline

**Figure 3:**
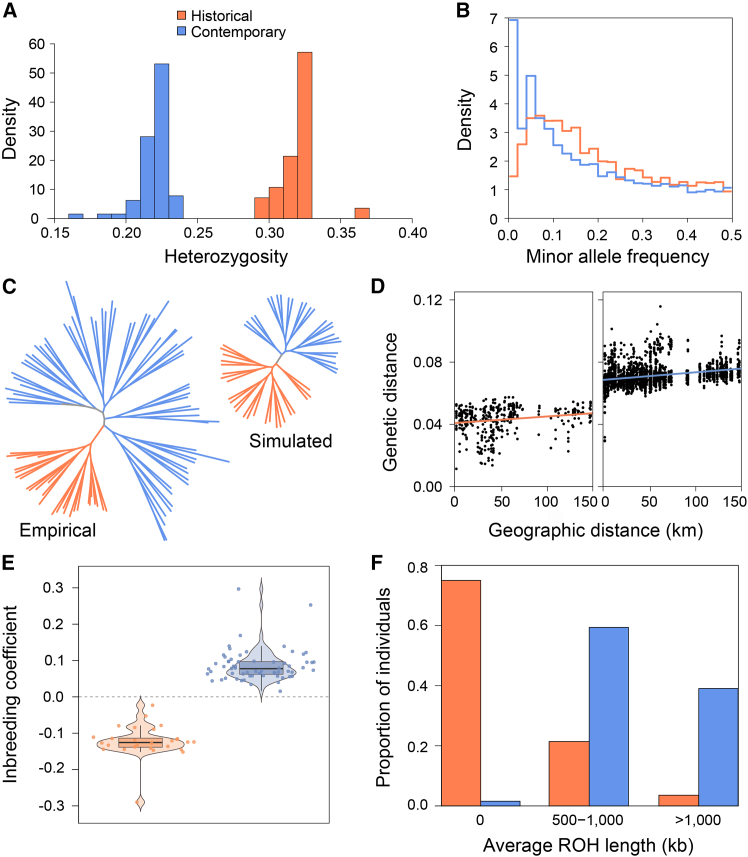
Genetic consequences of mid-20th century population decline (A) Distribution of heterozygosity in the historical and contemporary Marbled White samples. The medians are 0.32 and 0.22. The same color scheme is used across the entire graphic. (B) Minor allele frequency distribution in the historical and contemporary samples. (C) Unrooted maximum likelihood phylogenetic trees showing the evolutionary relationships among historical and contemporary individuals. The left tree is based on the empirical data from the matched localities. The right tree reflects simulated evolution over 75 generations at low population size (*n* = 2,000), starting from the empirically observed minor allele frequency distribution of the historical sample. (D) Relationship between genetic and geographic distance in the historical and contemporary samples. Each dot represents a pairwise comparison of two individuals. The lines are linear regression coefficients (slopes: 0.042 and 0.048). (E) Inbreeding coefficients for individuals from the historical and contemporary samples. Shown are the raw values and associated boxplots and density violins. (F) Distribution of the average length of runs of homozygosity (ROH) across individuals from the historical and contemporary samples.

Having observed a population decline in the Marbled White by more than 99% over two centuries, we next asked what genomic consequences such dramatic demographic change entails. To address this question, we first compared individual levels of genetic diversity, expressed by heterozygosity, between our historical and contemporary samples. This revealed a temporal decrease by approximately one third (31% decrease in median heterozygosity; Figure 3A).

To address the possibility that our genetic diversity estimates from the historical specimens might be inflated by changes in the DNA that occurred postmortem in the museum, such as base misincorporations resulting from cytosine deamination,^77^ we performed multiple robustness checks. These included the direct quantification of postmortem deamination, data re-analysis after the exclusion of all DNA positions prone to deamination, testing for a correlation between heterozygosity and sample age, and examining if a reduction in diversity was also observable at microsatellite loci unaffected by postmortem point mutations (Figures S3A–S3E). None of these analyses indicated artifacts, hence the massive difference in genetic diversity between our temporal samples can safely be ascribed to population size decline.

To obtain a more nuanced characterization of shifts in genetic variation beyond heterozygosity, we examined how the two temporal samples differed in the distribution of minor allele frequencies across SNPs. This showed that compared to the historical butterflies, the contemporary sample was strikingly enriched for invariant genomic positions (Figure 3B). This pattern is predicted for populations experiencing rapid and drastic reductions in effective population size, in which strong drift skews the frequency of genetic variants initially occurring in more balanced proportions.^78^^,^^79^^,^^80^

Observing marked shifts in genetic variation, we further asked what imprint strong genetic drift leaves on genetic relationships among individuals. We thus generated a phylogenetic tree by combining all historical and contemporary individuals, finding that the two temporal samples formed two mutually fully separated groups (Figure 3C; see Figure S9 for branch support). In addition, the terminal branches of the contemporary individuals proved substantially longer than those of the historical individuals (median increase: 30.9%; 95% confidence interval [CI]: 26%–44.5%; Figure S4). The specific tree topology (reciprocal monophyly) could be reproduced by simulating evolution under small population size over fewer than hundred generations when starting from the genetic variation contained in the historical sample (small tree in Figure 3C). (Because the simulations were initiated from the observed historical genetic variation, contemporary individuals could not exhibit private variants, precluding the emergence of longer terminal branches.) Together, these phylogenetic results suggest greater differentiation among contemporary than historical individuals due to strong drift, with some of this differentiation being shared among the contemporary samples and leading to their phylogenetic isolation from the historical ones. Accordingly, we predicted that for a given geographic distance between sampling localities, contemporary butterflies should be more genetically distinct than historical individuals; this difference in isolation by distance between the temporal samples was strikingly confirmed (Figure 3D).

Both the phylogenetic evidence of accelerated differentiation and the finding of more pronounced isolation by distance in the contemporary relative to the historical sample suggest that land-use intensification has caused evolution of the Marbled White to occur at a more restricted geographic scale. If so, this may imply an elevated probability of mating with relatives in small present-day populations, that is, inbreeding. To assess this possibility, we examined genome-wide inbreeding coefficients, where positive values reflect an excess of homozygosity. All contemporary individuals exhibited positive coefficients, consistent with inbreeding (Figure 3E). As a complementary analysis, we compared the occurrence of runs of homozygosity (ROH)—continuous chromosomal segments of homozygous genotypes typically arising from shared ancestry—between the individuals of our temporal samples. This revealed ROHs exceeding 500 kilobases in the vast majority of contemporary individuals (Figure 3F). A substantial fraction of individuals displayed ROHs >1 Mb, which is commonly interpreted as arising from recent inbreeding.^81^^,^^82^^,^^83^ The historical individuals, by contrast, exhibited mostly short ROHs.

### Contemporary genetic variation is not shaped by regional differences in land-use intensity

Our analyses so far focused on the comparison of butterfly sequence data across one and a half centuries characterized by drastic shifts in agriculture. However, these temporal changes in land use did not affect all areas within our study region equally. Notably, the Jura region situated in the north of Switzerland (Figure 1A) today still receives lower fertilizer and pesticide inputs and shows weaker landscape fragmentation and urbanization than the Central Plateau adjacent to the south.^84^^,^^85^^,^^86^^,^^87^ We took advantage of these regional differences in land use by looking for an associated difference in genetic variation. For this, we complemented our contemporary butterfly samples from the matched localities with contemporary individuals collected from numerous additional localities across the Jura region (*n* = 93 total individuals) and the Central Plateau (*n* = 155) (Figure 1; Table S1) that were also subjected to RAD sequencing.

**Figure 4:**
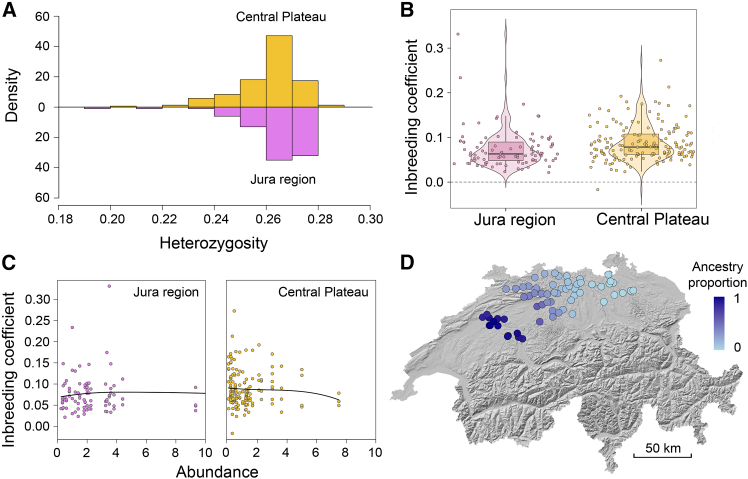
Regional genetic diversity, inbreeding, and population structure in contemporary butterflies (A) Distribution of heterozygosity among Marbled White individuals from the Jura and Central Plateau regions (medians: 0.27, 0.26). (B) Inbreeding coefficient for individuals from the two regions, visualized as in Figure 3E. (C) Inbreeding coefficient plotted against local butterfly abundance, quantified as individuals per minute, for the sampling locations in the two regions. The black lines represent the LOWESS smoothing fit. (D) Average ancestry proportion for all contemporary localities when assuming two genetic populations, the scenario best supported by the admixture analysis.

Performing field censuses, we observed approximately 3-fold larger populations of the Marbled White in the Jura region relative to the Central Plateau (Figure S5). However, levels of genetic diversity proved only trivially different between the two areas (Figure 4A). Similarly, although we detected indication of inbreeding in all but one individual (Figure 4B), the magnitude of inbreeding did not differ materially between the regions (median difference: 0.015; 95% CI: 0.005–0.024; Figure S6) and was not correlated with local abundance (Figure 4C). Our interpretation of this weak difference is that the relatively small present-day difference in land-use intensity between the Jura region and the Central Plateau is trivial in the light of the habitat losses caused by the mid-20^th^ century landscape transformation; compared to the historical situation, the current populations remaining in the Jura region are probably just minimally larger and not much less isolated than those from the Central Plateau. Dispersal at a local scale might be sufficient to prevent genetic differentiation among adjacent remaining habitat patches,^63^^,^^64^ but across our entire study region, drift might have become a key evolutionary factor. Supporting this view, an analysis of population structure^88^ across all our total contemporary localities identified substantial differentiation across the 150 km in east-west direction (Figure 4D), with the data best compatible with the presence of two distinct genetic groups (Figure S7). This observation reproduces a similar spatial trend found in an independent study of contemporary Marbled White across the same study region using microsatellite markers.^89^

Collectively, our comparison of contemporary butterflies between the Jura region and the neighboring Central Plateau indicates that current differences in land-use intensity between these areas have a minor effect on genetic diversity. However, generally low contemporary population size and population isolation appear to compromise population cohesion, resulting in genetic differentiation at the scale of our entire study region.

In conclusion, our results highlight the profound influence of human land use on a grassland specialist. Population size increased over centuries of traditional agricultural practice, followed by an abrupt decline within only a few decades. This trajectory exposes the two sides of human agricultural activity: moderate land use can promote abundance and diversity, whereas intensive management erodes both.

The observed trends in population size and associated genetic diversity can likely be generalized beyond the Marbled White to numerous formerly widespread and abundant grassland species now persisting in small, isolated remnant habitat patches within intensively managed landscapes. Many of these species are still classified as non-threatened, including the Marbled White, currently ranked as “least concern” in Switzerland and several other European countries.^90^^,^^91^ Yet, alongside ecosystem and species diversity, genetic diversity constitutes the third—and least appreciated—level of biodiversity. As the Marbled White strikingly exemplifies, dramatic biodiversity loss can occur without overt species extinction.

The loss of genetic diversity generally reduces evolutionary potential and hence the capacity to adapt to future environmental change. Our analyses further indicate that contemporary butterfly population sizes have dropped to levels at which inbreeding is becoming frequent, a process widely considered detrimental to organismal fitness. Whether current levels of inbreeding already impose a measurable fitness burden in the Marbled White remains unclear and cannot be resolved with genomic data alone; experimental information on organismal performance would be needed. However, genetic erosion lags behind contractions in population size^92^^,^^93^^,^^94^ and will likely intensify further in this butterfly’s future.

Prospects for reversing population and genetic decline appear limited. Human population size continues to increase both within our study region and globally, making a near-term reduction in land-use intensity and urbanization unlikely. A key unresolved question is whether the maintenance of traditionally managed habitats at current levels will suffice to ensure the long-term persistence of species that once benefited from moderate human land use.

### Limitations of the study

A limitation of this study is the restricted availability of historical museum specimens, which constrained both the number of individuals and the geographic coverage of sampling sites. Consequently, the historical dataset comprises only 29 individuals, in contrast to a substantially larger number of contemporary samples. In addition, the spatial distribution of historical specimens is uneven, with a strong bias toward western Switzerland and only limited representation from eastern Switzerland.

## Resource availability

### Lead contact

Further information and requests for resources should be directed to and will be provided by the lead contact, Daniel Berner (daniel.berner@unibas.ch).

### Materials availability

This study did not generate new unique reagents.

### Data and code availability

• All raw sequence data are available from the National Center for Biotechnology Information (NCBI) Sequence Read Archive (SRA) under BioProject accession number PRJNA1221805. Detailed accession information is provided in the supplemental material (Table S1).
• The SNP matrices underlying the primary analyses, along with the original code used for data analysis, have been deposited at Zenodo and are publicly available as of the date of publication. Accession numbers are listed in the key resources table.
• Any additional information required to reanalyze the data reported in this paper is available from the lead contact upon request.

## Acknowledgments

We kindly thank Hannes Baur for providing access to Marbled White specimens from the collection of the Natural History Museum Bern. Distribution data were obtained from info fauna, the national center for data and information on Swiss fauna, and from Gabrielle McLaughlin (Agroscope). Marius Roesti offered extensive technical support for RAD library preparation, and Lucas Blattner for laboratory procedures. Advice on whole-genome DNA library preparation was provided, and sequencing performed, by the Genomics Facility Basel, D-BSSE, ETH Zürich. Daniela Souza Costa aided the RELATE analysis. Computation was conducted at sciCORE, the scientific computing center at the University of Basel (http://scicore.unibas.ch/). Financial support was received from the 10.13039/501100001711Swiss National Science Foundation (grant 310030_200374 to D.B.). We also thank the three reviewers for their valuable suggestions on the manuscript.

## Author contributions

Study conception, D.B. and N.M.; field work, N.M. and D.B.; laboratory work, N.M. and D.B.; data analysis and interpretation, N.M. and D.B.; writing, N.M. and D.B.; funding, D.B.

## Declaration of interests

The authors declare no competing interests.

## Declaration of generative AI and AI-assisted technologies in the writing process

During the preparation of this article, the authors used ChatGPT to refine the phrasing of selected sentences. Following this assistance, the authors carefully reviewed and edited the content as necessary and assume full responsibility for the final published work.

## STAR★Methods

### Key resources table

**Table:** 

REAGENT or RESOURCE	SOURCE	IDENTIFIER
**Biological samples**		

289 butterfly specimens	This paper	Table S1

**Critical commercial assays**		

DNeasy Blood & Tissue kit	Qiagen	69506
Qubit fluorometer dsDNA high-sensitivity kit	Thermo Fischer Scientific	Q33232

**Deposited data**		

SNP matrices and original data	This paper	https://doi.org/10.5281/zenodo.19386897
Raw sequence data	This paper	NCBI-SRA: PRJNA1221805

**Software and algorithms**		

SAMtools v1.22	Li et al.^95^	https://www.htslib.org/
Rsamtools Bioconductor package v2.22.0	Morgan et al.^96^	https://bioconductor.org/packages/Rsamtools/
BCFtools v1.19	Danecek et al.^97^	https://www.htslib.org/
VCFtools v0.1.16	Danecek et al.^98^	https://doi.org/10.1093/bioinformatics/btr330
RELATE v1.2.1	Speidel et al.^65^	https://doi.org/10.1038/s41588-019-0484-x
GONE	Santiago et al.^76^	https://github.com/esrud/GONE
PLINK v1.9	Chang et al.^99^	https://www.cog-genomics.org/plink/1.9/
R package for approximate Bayesian computation (ABC)	Csillery et al.^100^	https://cran.r-project.org/web/packages/abc/index.html
IQ-TREE v2.3.6	Minh et al.^101^	https://iqtree.github.io/?utm_source=chatgpt.com
*ape* v5.3 R package	Paradis & Schliep^102^	https://cran.r-project.org/web/packages/ape/index.html
*phangorn* v2.5.5 R package	Schliep^103^	https://CRAN.Rproject.org/package=phangorn
ADMIXTURE v1.3.0	Alexander et al.^88^	https://doi.org/10.1101/gr.094052.109
PLINK 2 v2.00a3LM	Chang et al.^99^	https://doi.org/10.1186/s13742-015-0047-8
MapDamage 2.0	Jónsson et al.^104^	https://github.com/ginolhac/mapDamage

### Experimental model and study participant details

This study is based on wild, adult (imaginal), field-caught specimens of the butterfly *Melanargia galathea* (Linnaeus, 1758; Lepidoptera: Nymphalidae) (total *n* = 289). The dataset comprises 260 contemporary individuals collected in 2023 and 29 historical museum specimens collected between 1875 and 1955.

Contemporary specimens were collected using sweep nets, euthanized by freezing at −20°C, and stored in absolute ethanol until DNA extraction. Historical specimens were obtained from the Natural History Museum Bern, Switzerland. For transport to the University of Basel, the head and thorax of each pinned specimen were temporarily placed in absolute ethanol in individual 1.5 mL microcentrifuge tubes. No further treatment was performed prior to DNA extraction.

The study includes both male and female specimens, all belonging to the same generation. Because the analyses were based exclusively on autosomal DNA markers, with sex chromosome markers excluded, specimen sex does not influence the study outcomes.

Detailed information on all specimens, including their collection coordinates, sex, and NCBI Sequence Read Archive accession numbers, is provided in Table S1.

### Method details

#### Butterfly samples and study region

Our study focuses on a Western Palearctic grassland butterfly, the Marbled White (*Melanargia galathea,* Linnaeus, 1758; Lepidoptera: Nymphalidae), combining historical museum specimens with contemporary field-collected individuals from northern Switzerland (Table S1; Figure 1; map of study region created in QGIS v3.4.4.^105^). Historical samples (*n* = 29) consisted of pinned specimens from the collection of the Natural History Museum of Bern, collected between 1875 and 1955 from 18 localities (1–3 individuals per locality). Contemporary individuals were sampled using sweep nets during the species’ single annual flight period in June and July 2023, targeting four individuals from each of 64 localities (achieved at all but four sites; total *n* = 248). To minimize the demographic impact of specimen collection, our sampling was limited to a maximum of one female per site, with the remaining individuals being males. Sampling sites encompassed a range of grassland habitats, from railway embankments to large dry meadows, with a median distance of 5 km between each site and its nearest neighbor, and 104 km between the most distance sites. All specimens were preserved in absolute ethanol for subsequent genetic analyses.

For the 17 “matched” localities, both historical (*n* = 28; one historical individual was used only for inferring ancient population size) and contemporary (*n* = 64) specimens were available. The geographic distance between the precise capture sites of the two matching temporal samples never exceeded 1,000 m (mean = 538 m, minimum = 8 m). The remaining localities were selected to represent two adjacent regions differing in land-use intensity: the Jura region and the Central Plateau. Agricultural surface covers 42% of the Jura region compared to 49% of the Central Plateau, while urban environments account for 8% and 17%, respectively.^87^ Nitrogen deposition and pesticide use are also higher in the Central Plateau.^85^^,^^86^

To obtain a crude estimate of present-day population size at each locality, we recorded during field sampling the number of individuals observed within a radius of approximately 50 m around the locality coordinates over 1–30 min. These counts likely approached census numbers for small, isolated populations but provided noisier and likely downward-biased estimates for larger populations. This limitation in mind, we expressed local abundance for each locality as individuals per minute.

#### Generation of sequence data

DNA was extracted from all 289 individuals from the thorax and head using the DNeasy Blood & Tissue Kit (Qiagen, Hilden, Germany), with elution into 100 μL low-EDTA TE buffer.

DNA concentrations were measured with a Qubit 3.0 Fluorometer (Thermo Fisher Scientific, USA) using the dsDNA High Sensitivity Assay Kit, indicating a mean concentration of 17.2 ng/μL (sd: 7.9) for the historical samples and 29.6 ng/μL (sd: 5.7) for the contemporary ones. For the up to 150 years old historical samples, we explored the DNA fragment length distribution for eight individuals (collected 1880–1950) using a Fragment Analyzer. Given consistently strong fragmentation of these samples (peak length 80–110 bp), DNA from all historical specimens was ligated to Illumina adapters and amplified in five PCR cycles. Libraries were then subjected to a 1.4× SPRI clean-up to remove short fragments and adapter dimers, resulting in fragment length peaks around 200 bp. The historical specimens were then whole-genome sequenced in paired-end 100 bp mode without further PCR enrichment on an Illumina NovaSeq 6000 S4 flow cell, yielding an average read depth of 36x (range: 9x - 81x; Table S1).

The contemporary samples were subjected to restriction site-associated DNA (RAD) library preparation using the PstI restriction enzyme, which targets about 107,000 recognition sites across the 600 Mb Marbled White genome. This followed classical protocols,^106^^,^^107^ except that library-specific P1 adapters were designed to index individual libraries for joint sequencing. Final library amplification was performed in 10 replicate PCRs with 19 cycles to minimize amplification bias. The RAD libraries were sequenced in paired-end 100 bp mode on an Illumina NovaSeq 6000 S4 flow cell to an average read depth of 35x (range: 7x −117x; Table S1). Only the R1 reads (including the restriction site residual) were used for analysis.

Raw reads were inspected with FastQC v0.12.1^108^ for sequence quality, GC content, duplication levels, and adapter contamination. The relatively short insert sizes of the historical samples made residual Illumina adapter trimming necessary, which was performed using Cutadapt v4.6^109^ and confirmed with FastQC.^108^ All reads were then aligned to the Marbled White female reference genome (NCBI Assembly: GCA_920104075.1, ilMelGala2.1) using BWA-MEM2 v2.2.1.^110^^,^^111^ Mapping quality and alignment statistics were assessed with SAMtools v1.22.^95^ BAM files were generated and sorted using SAMtools v1.22^95^ and the Rsamtools Bioconductor package v2.22.0.^96^

#### Identification of genetic markers and filtering

Single-nucleotide polymorphisms (SNPs) were called separately for three datasets: (i) a matched localities dataset combining both historical and contemporary individuals from the 17 matched localities (total *n* = 92; 28 historical, 64 contemporary), (ii) a contemporary-only dataset (*n* = 248; 93 Jura, 155 Central Plateau), and (iii) a historical-only dataset (*n* = 29). Variant calling was performed for each dataset using the mpileup and call functions in BCFtools v1.19.^97^ Raw variant calls were subsequently filtered using VCFtools v0.1.16.^98^ Filtering retained only autosomal, biallelic SNPs; indels were excluded. Additional filtering criteria included a minimum sequencing depth of 10x and a maximum depth of 150×, a minimum Phred-scaled quality score of 30, and genotype calls present in at least 90% of individuals for the matched localities and at least 95% for the contemporary-only and historical-only datasets. A minor allele frequency (MAF) filter (≥0.03) was applied only to the contemporary-only and matched localities datasets.

After filtering, the matched localities dataset comprised 51,379 SNPs and was used for all analyses comparing pre- and post-intensification genetic patterns. The contemporary-only dataset retained 69,261 SNPs and was used for comparing the Jura region and Central Plateau. The historical-only dataset retained 352,578 SNPs and was used exclusively for estimating ancient effective population size.

#### Ancient population size

To explore demographic trends of the Marbled White thousands of years before agricultural intensification, we inferred ancient effective population sizes (Ne) using RELATE v1.2.1^65^, which reconstructs historical Ne trajectories based on the coalescence of alleles, with highest reliability between approximately 1,000 and 100,000 generations before present (corresponding to years in this species). This was restricted to the historical-only dataset because we feared that the massive mid-20^th^ century population size decline could render the contemporary samples unreliable for demographic inference far back in time, and because only the historical individuals were whole-genome sequenced, thus producing more than five times higher SNP resolution compared to the other datasets.

The analysis involved phasing genotypes into haplotypes using PLINK v1.9.^99^ The phased variant data, filtered as described above, were converted into RELATE’s haplotype (.haps) and sample (.sample) formats using the RelateFileFormats module. SNP annotations (.annot) were generated with ancestral alleles inferred from the reference genome. Chromosome-specific recombination maps were created assuming a homogeneous recombination rate of 5.5 cM/Mb, based on the butterfly *Heliconius* m*elpomene.*^112^ Genealogical inference was performed with the RELATE module in “All” mode, which requires specification of an Ne to scale coalescent times. We therefore supplied a constant Ne of 200,000 as an initial scaling parameter and a mutation rate of 2.8 × 10^−9^ per site per generation, the latter again estimated from *Heliconius.*^113^ Population size history was then estimated using the EstimatePopulationSize script, for one generation per year and 23 chromosomes. Assuming initial effective population sizes between 10,000 and 400,000 had only a minor effect on the inferred demographic trajectory up to ∼25kya back in time, although more pronounced differences were observed in the deeper past. However, given our interest in potential demographic consequences of human activity, we refrained from interpreting demographic inference more than c. 20,000 years into the past.

The stability of the estimates was quantified by different approaches, including jackknifing (randomly subsampling 20 of the 29 individuals for ten estimation runs), inferring Ne for each chromosome separately, or randomly resampling 75% of all SNPs per chromosomes for each of 20 replicate estimation runs. All these methods indicated high estimation stability (Figure S8), hence we expressed uncertainty around the temporal point estimates by the estimation range across the jackknife replicates.

#### Recent population size

Our main approach involved estimating recent changes in effective population based on linkage-disequilibrium using GONE,^76^ which provides reliable Ne estimates over the last 200 generations. Analyses were conducted on the matched localities dataset. Input files (.map and .ped formats) were generated separately for historical and contemporary samples using PLINK v1.9.^99^ Based on these files, GONE analyses were conducted for both temporal samples with default parameter settings. To account for stochastic variation, we ran 40 independent replicates per dataset and report the mean Ne trajectory across replicates.

To validate the robustness of the strong population decline inferred by GONE for the contemporary sample based on linkage disequilibrium among pairs of loci, we developed an alternative, methodologically distinct estimation approach combining forward simulations and Approximate Bayesian Computation (ABC).^100^ Details are provided in Figure S2, but in brief, we here started from the SNP matrix for the historical individuals to derive their MAF distribution across all SNPs (see below; visualized in Figure 3B). Based on this MAF distribution, we simulated genetic drift across 75 generations (approximating the period from 1950 to 2023) across a broad range of population sizes. For each simulated population size, we recorded the resulting MAF distribution after evolution. Finally, we derived the MAF distribution from the SNP matrix of the contemporary samples, and used ABC to explore which population sizes resulted in simulated MAF spectra most closely approximating their empirically observed counterpart.

#### Genetic diversity

As individual-level measure of genetic diversity, we used the observed heterozygosity, obtained by dividing the number of heterozygous positions by the total number of positions genotyped in a given individual.

An alternative, more comprehensive population-level comparison of genetic diversity between the temporal samples was conducted based on the MAF distribution, summarizing the distribution of allele frequencies across loci and offering qualitative insights into population history. MAF calculation was based on the matched localities dataset, and considered only SNPs represented by at least 20 diploid genotypes in either temporal sample (i.e., 40 haploid alleles). For the 50,601 SNPs passing this filter, the folded MAF distribution was generated separately for the historical and contemporary samples. For visualization, MAFs were binned into 25 classes of width 0.02.

#### Phylogenetic tree

A maximum-likelihood phylogenetic tree was generated with IQ-TREE v2.3.6^101^ based on the SNPs from the matched localities dataset, using a general time-reversible (GTR) substitution model with ascertainment bias correction. Branch support was evaluated using 10,000 bootstrap replicates (presented in Figure S9). Differences in mean branch lengths between the temporal samples were quantified by extracting terminal branch lengths from the treefile produced by IQTREE. To quantify the precision of the point estimate, we used a bootstrap resampling strategy: for each of 10,000 iterations, terminal branches were chosen at random with replacement from the tree for the historical samples and contemporary samples, and the median difference between contemporary and historical branch lengths was calculated. The 95% compatibility interval was then determined based on the 0.025 and 0.975 quantiles of the bootstrap distribution of the median differences.

To assess the robustness of the inferred tree topology and to exclude potential technical biases, we repeated the generation of the tree with several modifications, including: (i) applying a more stringent minor allele frequency (MAF) filter (0.05); (ii) reducing sample size of the contemporary butterflies to the exactly same number as for the historical ones (*n* = 28); (iii) excluding individuals with low read depth (<10x, five historical individuals); and (iv) restricting the dataset to positions without any missing data. None of these approaches altered the overall tree topology.

Finally, we used forward simulations to evaluate whether the reciprocal monophyly between the historical and contemporary samples observed in our phylogeny could plausibly be attributed to strong drift following massive population size reduction. As a starting point, the simulations used the empirically observed MAF distribution of the historical sample (see above). From this distribution, we derived multi-locus genotypes for 25 diploid individuals by randomly sampling allele pairs with replacement from each SNP. These genotype data were concatenated within each individual, and the resulting multi-locus genotypes written into a FASTA file. The population was then allowed to evolve over 75 generations at a constant haploid population size of 2,000, 5,000, 10,000, 20,000 or 50,000. The SNPs were assumed to be unlinked, and allele frequencies in each generation were obtained by binomial sampling from parental allele frequencies. After evolution, a second set of 25 diploid individuals was generated using the procedure described, and appended to the above FASTA file for subsequent phylogenetic tree construction. For this, we here used the R packages *ape* v5.3^102^ and *phangorn* v2.5.5.^103^ Using the best-fitting substitution model (JC + G), maximum-likelihood trees were inferred and visualized (Figure S10). Analyses were repeated in 2–3 replicates for each population size, producing results qualitatively consistent among the replicates (the first replication made for each sample size was chosen for presentation). Using a simple neighbor-joining approach instead of maximum-likelihood for tree generation led to the same conclusions.

Note that these simulations explored tree topology but were not expected to reproduce branch length differences between the temporal groups; the simulations were conditioned on the initial MAF distribution of the historical sample, hence the evolved population could not exhibit private SNPs. The latter, however, occurred in the empirical contemporary sample, mainly because of limited sampling of the historical butterflies (missed alleles; we consider *de novo* mutation in the contemporary sample less important, given the short time period and the small mutation target provided by the small contemporary population).

#### Isolation by distance

To quantify pairwise genetic distances among individuals, we concatenated genotypes across all SNPs in the matched localities dataset to FASTA. Distances were then calculated separately for the historical and the contemporary sample using the dist.ml function in the R package *phangorn* v2.12.1^103^ Pairwise geographic distances between sampling localities were calculated from latitude and longitude coordinates. Combined, these data allowed us to assess how strongly genetic distance was related to geographic distance.

#### Inbreeding

Individual inbreeding coefficients (F_HOM_) were calculated from the matched localities dataset as well as from the contemporary-only dataset. Inbreeding coefficients were calculated using PLINK v1.9^99^ by using the --het function. Positive F_HOM_ values indicate excess homozygosity relative to Hardy–Weinberg expectations, consistent with inbreeding, whereas negative values reflect excess heterozygosity, potentially due to outbreeding or population expansion in the past. We calculated the difference in median F_HOM_ between the Jura region and the Central Plateau, estimating uncertainty by bootstrapping. For this, we took 10,000 F_HOM_ resamples within each population, recalculated population medians, and recorded their difference (Central Plateau – Jura region). The 95% compatibility interval was derived from the quantiles of the resulting bootstrap distribution (see above). Potential relationships between individual F_HOM_ and local abundance were visualized with scatterplots and LOWESS smoothing for the Jura region and the Central Plateau separately.

Runs of homozygosity (ROH) were investigated using PLINK’s --homozyg function. ROHs were inferred using the following parameters: a minimum ROH length of 500 kb (-homozyg-kb 500); at least 50 SNPs per ROH (--homozyg-snp 50); a maximum gap of 1,000 kb between adjacent SNPs (--homozyg-gap 1000); a minimum SNP density of one SNP per 50 kb (--homozyg-density 50); tolerating no more than one heterozygous site per window (-homozyg-window-het 1) and up to five missing genotypes per window (--homozyg-windowmissing 5); and a sliding window size of five SNPs (--homozyg-window-snp 5).

#### Ancestry inference

Population structure and individual ancestry proportions in the contemporary sample were examined using ADMIXTURE v1.3.0^88^. Input files were created by converting variant call format (VCF) files to binary BED format using PLINK 2 v2.00a3LM^99^ (--make-bed). Pre-specified population numbers (K) from 1 to 10 were considered. The optimal K was determined using ADMIXTURE’s cross-validation procedure, selecting the model with the lowest cross-validation (CV) error.

#### Checks for indications of analytical bias due to postmortem DNA degradation

Because DNA degrades over time, historical samples can exhibit postmortem damage. The main concern to our study was cytosine deamination, resulting in spurious C to T and G to A substitutions.^77^ Such artifacts can, for instance, inflate estimates of genetic diversity. We addressed this possibility through five robustness checks.

First, we used MapDamage 2.0^104^ to test for overrepresentation of A and T substitutions indicative of deamination in the historical BAM files.^114^ No such bias was detected (Figure S3A).

Second, we reasoned that if our polymorphisms were at least partly artifacts from deamination, SNPs private to the historical sample (i.e., positions polymorphic only in the historical sample but monomorphic in the contemporary one) should be enrichment for C/T and G/A SNPs relative to the baseline provided by the SNPs private to the contemporary sample. This was evaluated based on the matched localities dataset, revealing a minute difference in the proportions of potentially deamination-associated polymorphisms between the two temporal samples, fully compatible with the absence of artifacts (Figure S3B).

Third, if postmortem degradation influenced genetic diversity, the latter should increase with sample age, given our historical samples covered eight decades. We thus looked for a possible relationship between heterozygosity and age in this sample, but found no indication of such a trend (Figure S3C).

Fourth, we repeated both the comparison of heterozygosity and the construction of the phylogenetic tree using just the subset of SNPs in which all C/T and G/A polymorphisms were removed (18,497 out of the 51,379 SNPs in the matched localities dataset). These analyses produced quantitatively similar results supporting the same results as with the full data (Figure S3D).

Finally, our fifth robustness check examined genetic diversity at microsatellite loci. Because microsatellite mutations arise from changes in tandem repeat length and are not expected to be affected by DNA deamination, they provide an estimate of genetic diversity independent of SNP-based measures. We therefore compared microsatellite diversity between temporal samples from matched localities, expecting reduced diversity in contemporary individuals. To identify suitable microsatellites, we manually screened RAD loci in BAM files from five randomly chosen contemporary individuals for various di- and trinucleotide motifs showing at least seven repeats. As butterfly genomes often contain microsatellite families generated by copy-transposition,^89^^,^^115^^,^^116^ we retained only loci showing no variation in the 15 bp flanking region to either side and exhibiting typical read depth, ensuring uniqueness and homology. We further required loci to be present in at least 18 individuals per temporal sample, yielding seven loci for analysis. Although a limited number, this still allowed an assessment of trends. Individuals were the genotyped by eye based on repeat number. To avoid ambiguity in zygosity in cases of low read depth, we randomly sampled only one allele per individual for diversity analyses. Allelic richness and Shannon diversity were then calculated separately for the historical and contemporary butterflies. This procedure was repeated across 10,000 replicates, and differences in mean diversity between the temporal samples were plotted for each locus and metric. The results qualitatively supported our SNP-based findings, with the contemporary sample showing lower allelic richness and Shannon diversity than the historical one (Figure S3E). The chromosomes and alignment positions of the RAD loci harboring the microsatellites are: M1: OV049859.1_15684248; M2: OV049855.1, 40427397; M3: OV049856.1, 12950729; M4: OV049857.1, 762903; M5: OV049863.1, 6742488; M6: OV049876.1, 2479973; M7: OV049865.1, 19452047.

We further note that our investigation used the same sequencing strategies and marker generation pipeline as Blattner et al.,^117^ a study also integrating sequence data from decades-old museum and contemporary samples. That study confirmed that the combination of historical whole-genome data with contemporary RAD sequencing data supports robust genomic inference.

### Quantification and statistical analysis

All sample sizes underlying our analyses can be found in the corresponding sections of the Method details. The study avoids significance testing and instead quantifies estimation precision by bootstrapping and jackknifing, as also specified in the Method details. Unless stated otherwise, all analyses and visualization were performed with the R language (v4.5.2).

## Supplemental information

Document S1. Figures S1–S10

Table S1. Sample descriptions
