# Supplementary material for: Historical and contemporary butterfly genomes reveal genetic erosion in response to land-use intensification
Source: iScience. 2026 Jul 22;29(8):116884. doi: 10.1016/j.isci.2026.116884 (PMC13426219; doi:10.1016/j.isci.2026.116884)
Supplement: Document S1. Figures S1–S10 [file mmc1.pdf]

iScience, Volume *29*

Supplemental information

Historical and contemporary butterfly genomes reveal genetic erosion in response to land-use intensification

Nathalie Matthey-de-l'Endroit and Daniel Berner

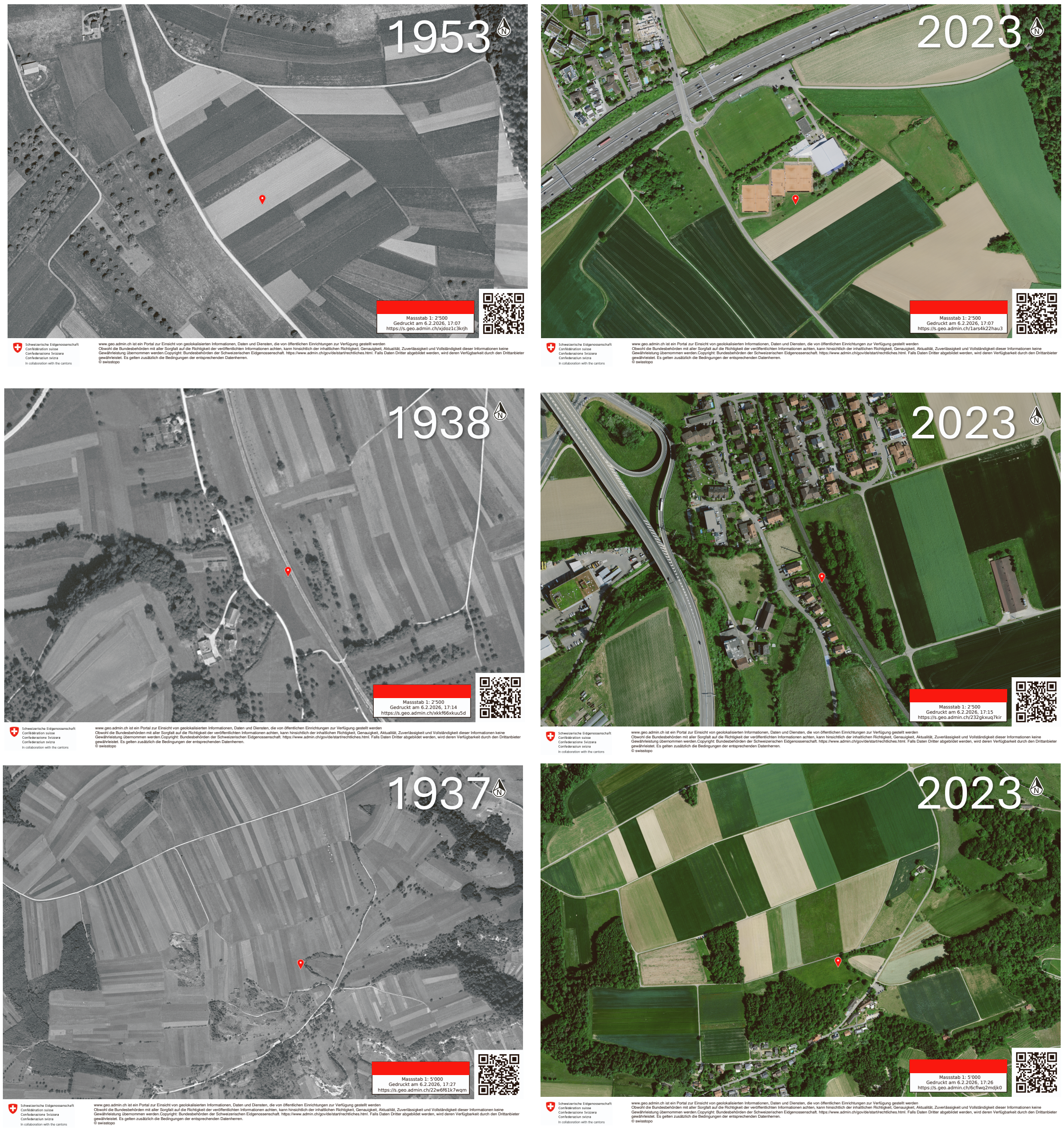

Figure S1. Aerial images of three exemplary matched localities showing historical and contemporary conditions in roughly the years of specimen collection (from top to bottom localities ZH2, FR1 and FR3), Related to Figure 1. Red flags represent the coordinates where contemporary individuals were sampled in summer 2023. The images illustrate some characteristic elements of land-use intensification, including the increase in field (parcel) size facilitating mechanical cultivation, and the expansion of settlement area and human infrastructure into semi-natural environments. A key aspect not revealed by the images but crucial to the present study is the boost in grassland productivity achieved by the application of synthetic fertilizer, causing major changes in microclimate and allowing for a higher mowing frequency.

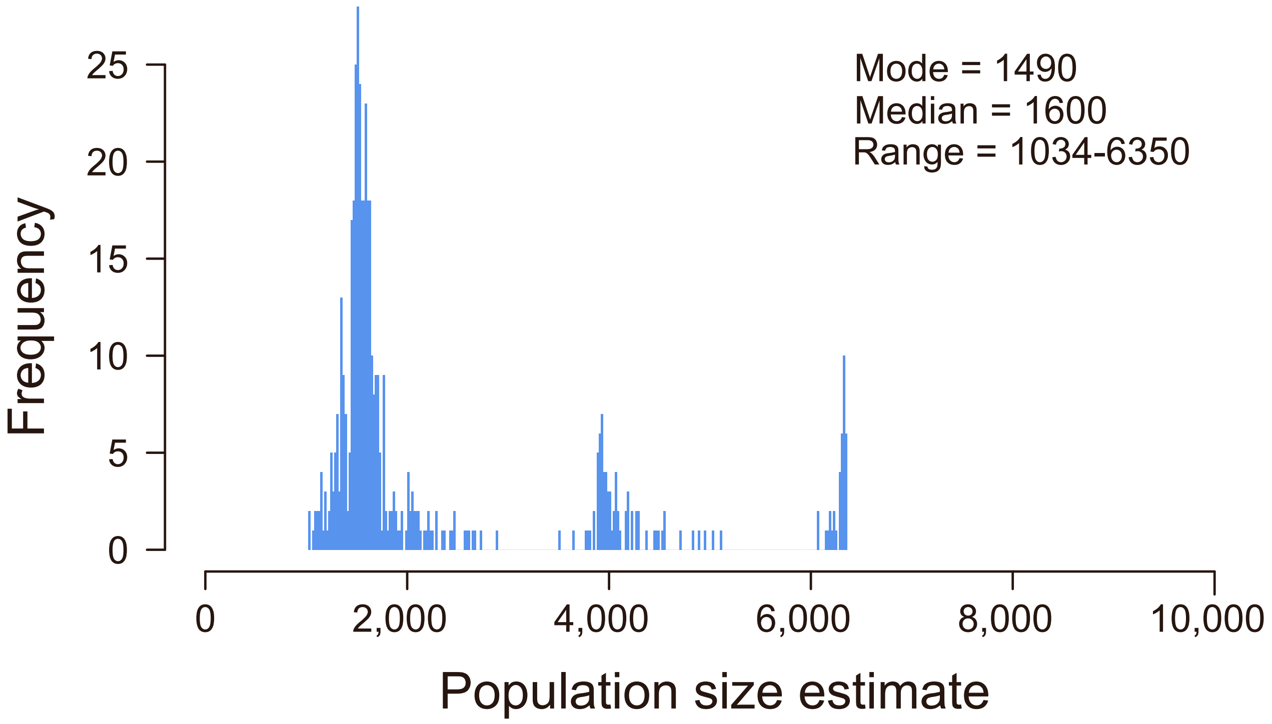

Figure S2. Estimation of contemporary population size by simulations and Approximate Bayesian Computation (ABC), Related to Figure. 2B and Figure 3B. ABC-estimation of the haploid size of a population required to produce a minor allele frequency (MAF) distribution approximating the empirically observed contemporary MAF distribution when evolving from the MAF distribution observed in the historical samples (see Figure. 3B). The underlying simulations (10,000 replicates) considered drift as the only process influencing allele frequencies; assumed evolution over 75 generations, approximating the period from 1950 to 2023 (the Marbled White has one generation per year); involved binomial sampling of alleles from one generation to the next at each SNP; assumed unlinked loci; and drew population sizes with uniform probability across the range from 400 to 20,000 haploid individuals (preliminary runs aimed at narrowing the relevant search range considered population sizes up to 600,000). After evolution, the MAF distribution across all SNPs was determined, folded, and binned with a 0.02 interval width as described for the empirical MAF spectra. ABC parameter search was based on the full binned MAF spectra. The histogram and the parameter estimates given are derived from 500 ABC estimation runs performed by using the abc R package v2.2.2[1] with a neural network approach and a tolerance of 0.1. Alternative tolerance values were also explored and yielded similar results supporting the same qualitative conclusions. From each ABC run, we retained the median population size estimate for presentation. Note that all estimates are well below 10,000 individuals, supporting the extremely small butterfly population size also indicated by our main analysis based on a methodology different approach (GONE[2]).

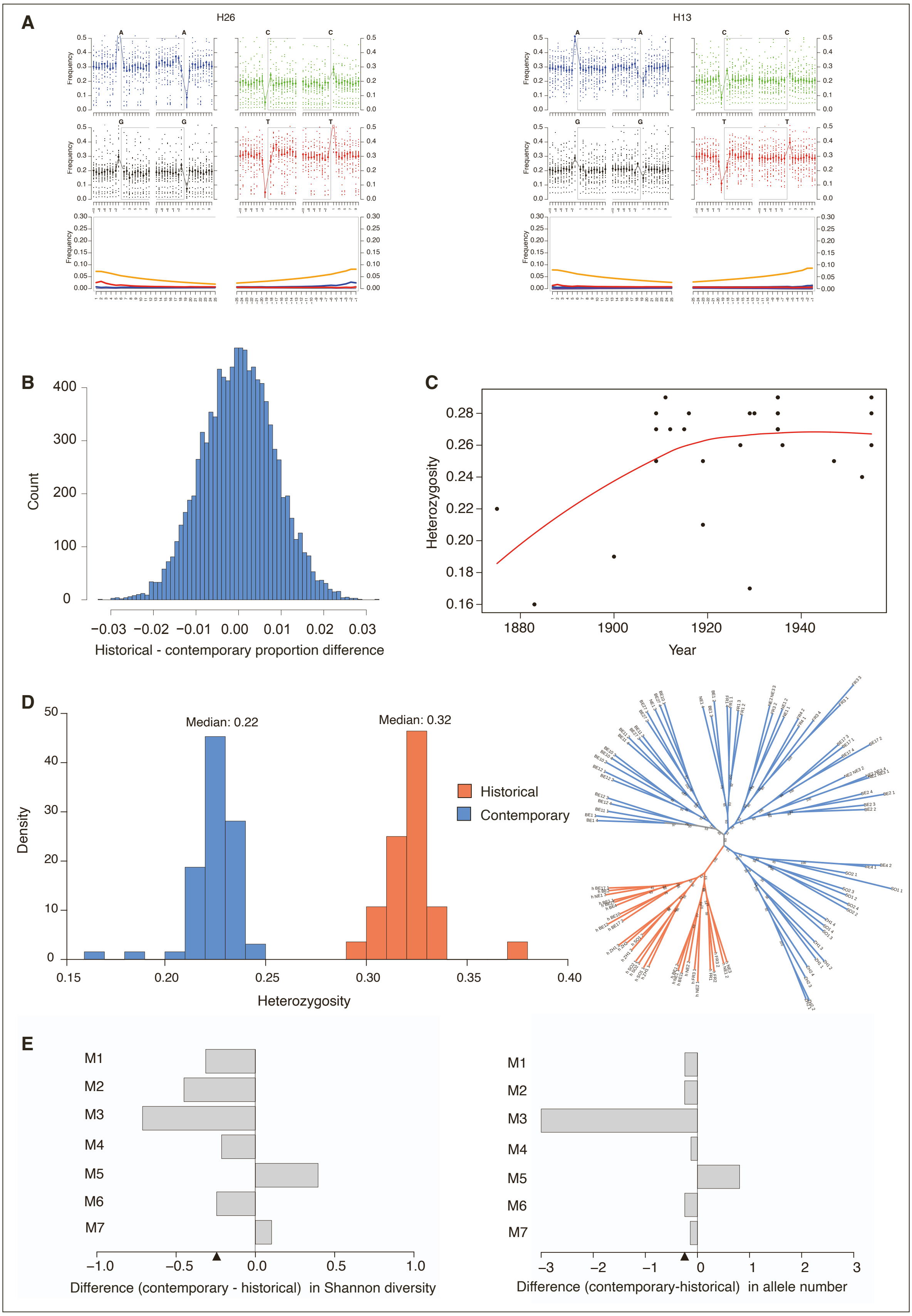

A
H26
H13
Frequency
B
Count
Historical - contemporary proportion difference
C
Heterozygosity
Year
D
Median: 0.22
Median: 0.32
Historical
Contemporary
Density
Heterozygosity
E
M1
M2
M3
M4
M5
M6
M7
Difference (contemporary - historical) in Shannon diversity
Difference (contemporary-historical) in allele number

Figure S3. Robustness checks confirming the absence of analytical artifacts due to post-mortem DNA damage, Related to Figure 3.

A) Representative *MapDamage*[3] output for the oldest (left; H26, 1875) and youngest (right; H13, 1955) historical samples. The four upper panels show the base composition within the sequence read region (open grey box indicates read boundaries) and the flanking region of the read. The lower panels display position-specific substitution patterns from the 5′ end (left) and the 3′ end (right) of the reads. The color scheme is red for C to T substitutions; blue for G to A substitutions; grey for all other substitution types; and orange for soft-clipped bases. No enrichment of A or T is observed, indicating the absence of spurious deamination-induced mutations in either sample.

B) Distribution of the difference between the historical and the contemporary sample in the proportion of potential polymorphisms originating from post-mortem deamination (i.e., C to T and G to A) among the total SNPs private to each temporal sample. The distribution is based on 10,000 bootstrap resampling iterations for the historical (n = 6,188) and the contemporary (n = 984) private SNPs, with the point estimates for the proportions being 0.497 and 0.482. The bootstrap distribution peaks at zero difference, thus providing no indication of an enrichment for deamination SNPs in either sample.

C) Heterozygosity plotted against sample age for all historical individuals. Across the time range well covered by data, there is no trend of increasing of heterozygosity with age, and the oldest individuals do not exhibit the highest heterozygosity. There is thus no indication of spurious polymorphisms from post-mortem degradation.

D) Heterozygosity distribution (left) and unrooted maximum likelihood phylogenetic tree (right) based on a filtered dataset in which the SNP classes potentially affected by deamination (18,497 out of 51,379 SNPs) were excluded. Note the consistency with Figures 3A and 3C in the main paper.

E) Difference in Shannon diversity and allelic richness across seven microsatellite loci located on RAD sequences. For both metrics, a single allele per individual and temporal sample was drawn at random 10,000 times, and the historical sample minus contemporary sample difference calculated for each replicate. The values shown represent the means of each distribution, with negative values indicating a loss of genetic diversity – the trend suggested by these data. Black triangles denote the median values across loci. The microsatellites are located on RAD sequences with the following alignment chromosomes and positions: M1: OV049859.1_15684248; M2: OV049855.1, 40427397; M3: OV049856.1, 12950729; M4: OV049857.1, 762903; M5: OV049863.1, 6742488; M6: OV049876.1, 2479973; M7: OV049865.1, 19452047

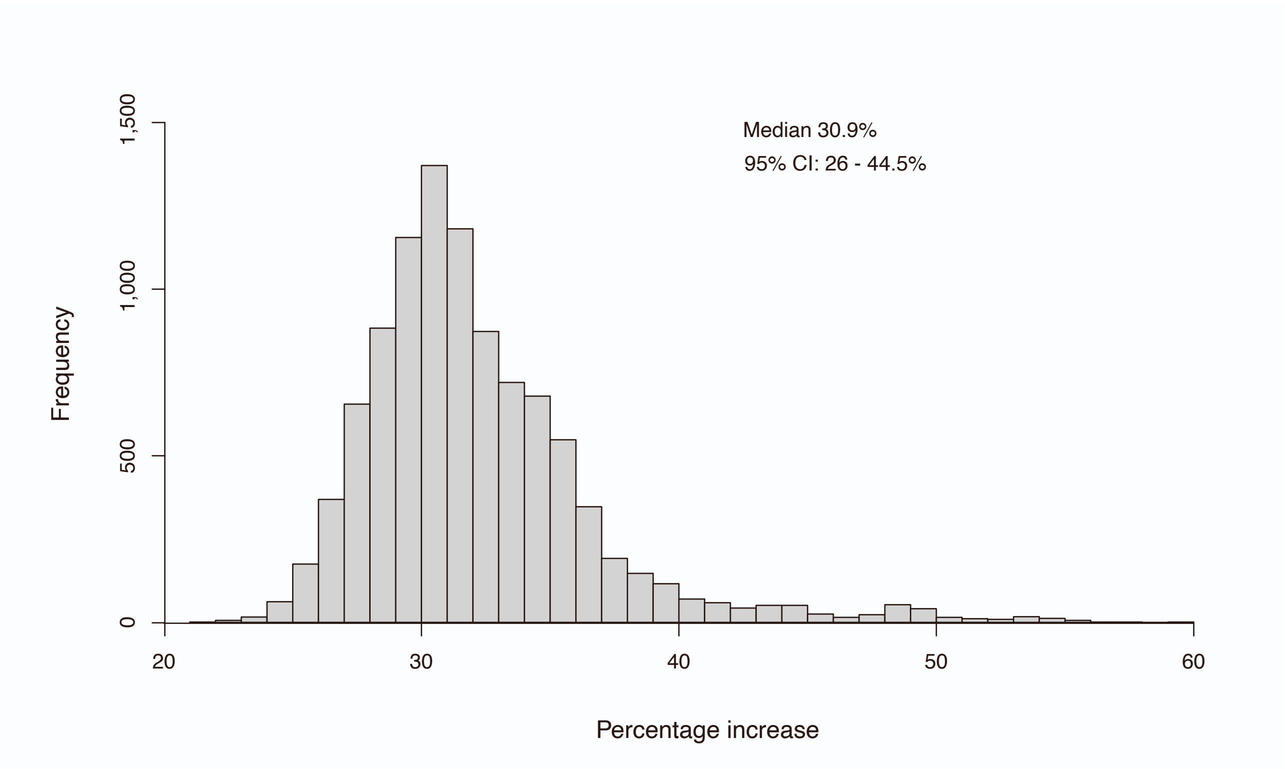

Figure S4. Bootstrap distribution of the percentage increase in terminal branch length in contemporary compared to historical individuals, Related to Figure 3C. The distribution was obtained by bootstrapping each group 10,000 times and recalculating the contemporary-historical difference in median branch length for each iteration.

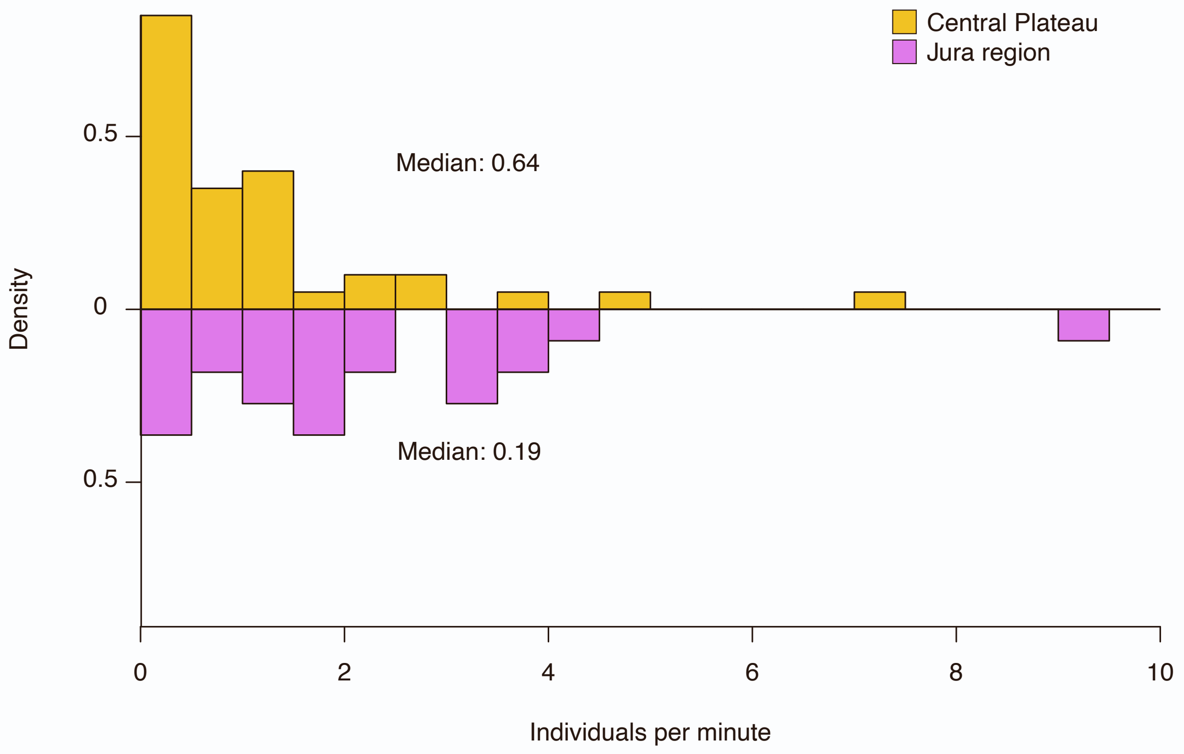

Figure S5. Local abundance of the Marbled White in the Central Plateau and the Jura region, Related to Figure 4C. Abundance estimated as individuals per minute (n = 93 and n = 155 locations for the Jura region and the Central Plateau). To facilitate visualization, one extreme outlier locality from the Jura region (54 individuals per minute) was excluded from the graphic. Median local abundance in the Jura region is three times as high as in the Central Plateau.

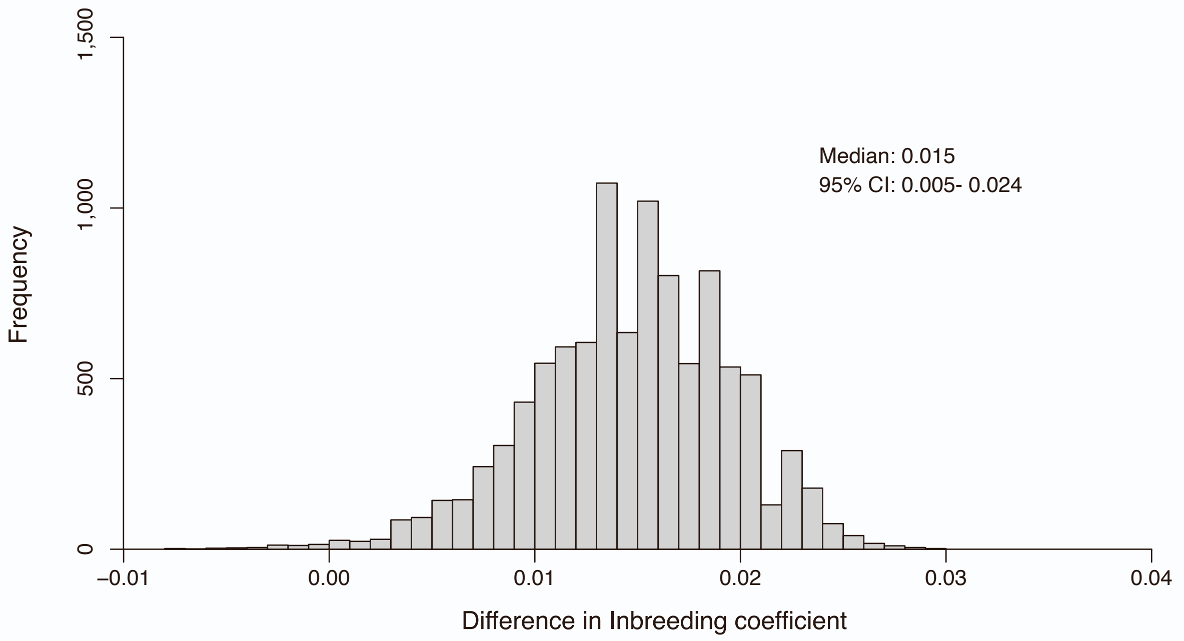

Figure S6. Difference in median inbreeding coefficient between the Central Plateau and the Jura region, Related to Figure 4B. Shown is the bootstrap distribution based on 10,000 resamples, each time recalculating the median difference (Central Plateau minus Jura region). The values given represent the empirically observed point estimate and the associated 95% compatibility interval, the latter expressed as the 0.025 and 0.975 percentiles of the bootstrap distribution. Although the distribution is well compatible with stronger inbreeding in the Central Plateau than the Jura, the magnitude of this difference is very low – less than 10% of the estimated difference between the historical and the contemporary sample (Figure 3E).

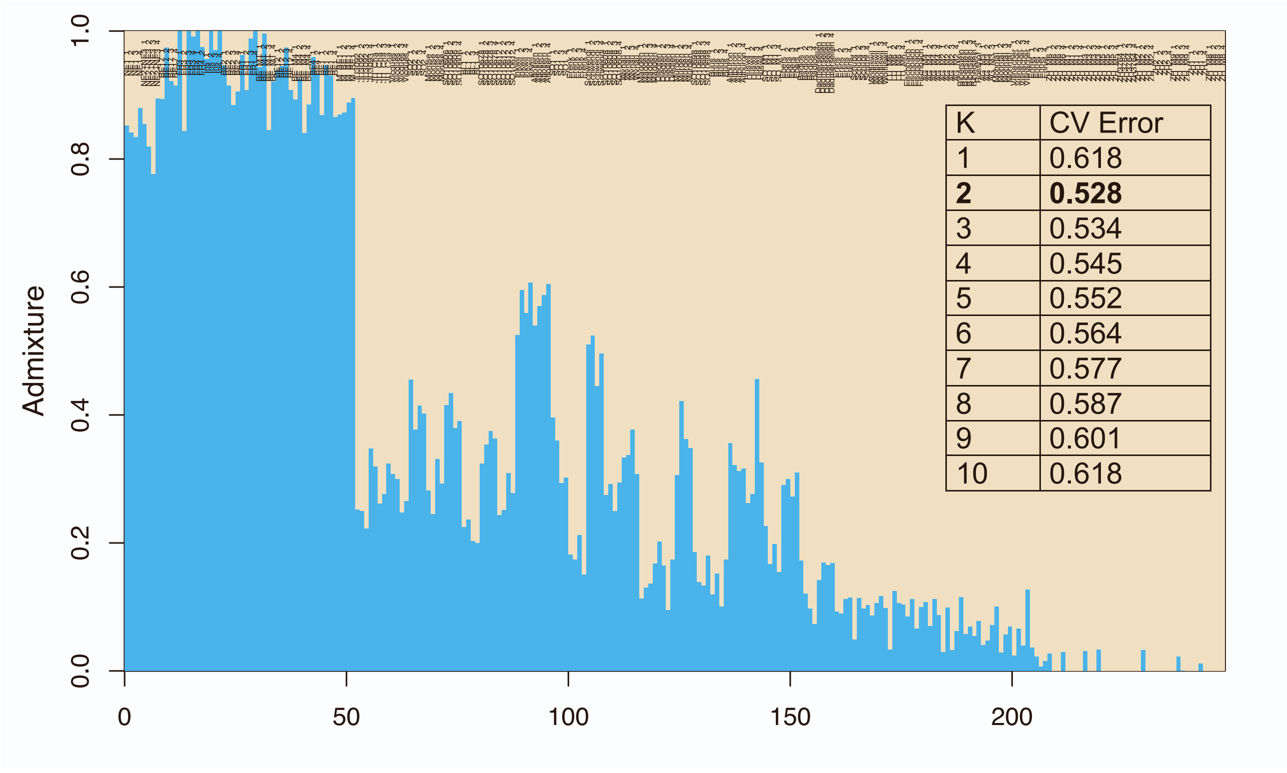

K	CV Error
1	0.618
**2**	**0.528**
3	0.534
4	0.545
5	0.552
6	0.564
7	0.577
8	0.587
9	0.601
10	0.618

Figure S7. Ancestry inference with ADMIXTURE, Related to Figure 4D. Population ancestry proportions for all contemporary individuals *(n* = 248), shown as vertical bars and ordered by longitude for the most likely scenario of two genetic populations (K = 2). Sample names are indicated above each bar. The insert table presents the cross-validation (CV) error from ADMIXTURE analyses assuming up to ten populations (K = 1-10). Lower CV error indicates better model fit (best fit printed bold).

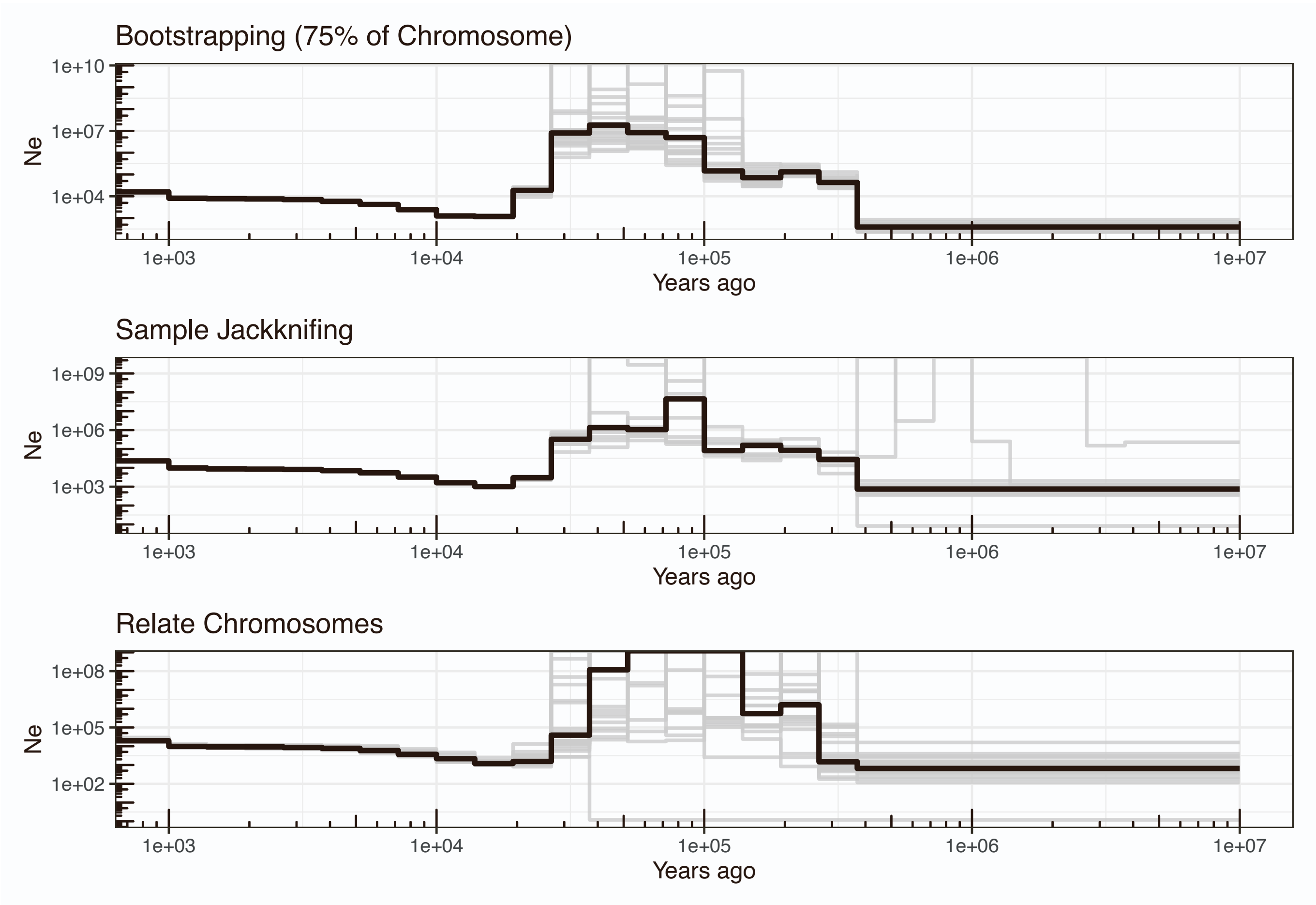

Figure S8. Robustness of effective population size (Ne) estimates inferred with RELATE under alternative resampling strategies, Related to Figure 2A. Top: performing 20 independent estimation runs, each based on a random resampling of 75% of SNPs per chromosome middle: jackknife resampling by randomly subsampling 20 out of the 29 individuals across 10 estimation runs; and bottom: separate Ne inference for each chromosome individually, treating the chromosomes as replicates. Black lines represent median population size across the corresponding replicates. All these approaches confirm high robustness of the Ne estimation from the postglacial minimum toward the present – the focal time range in the present study, although estimates deeper backward in time are less stable.

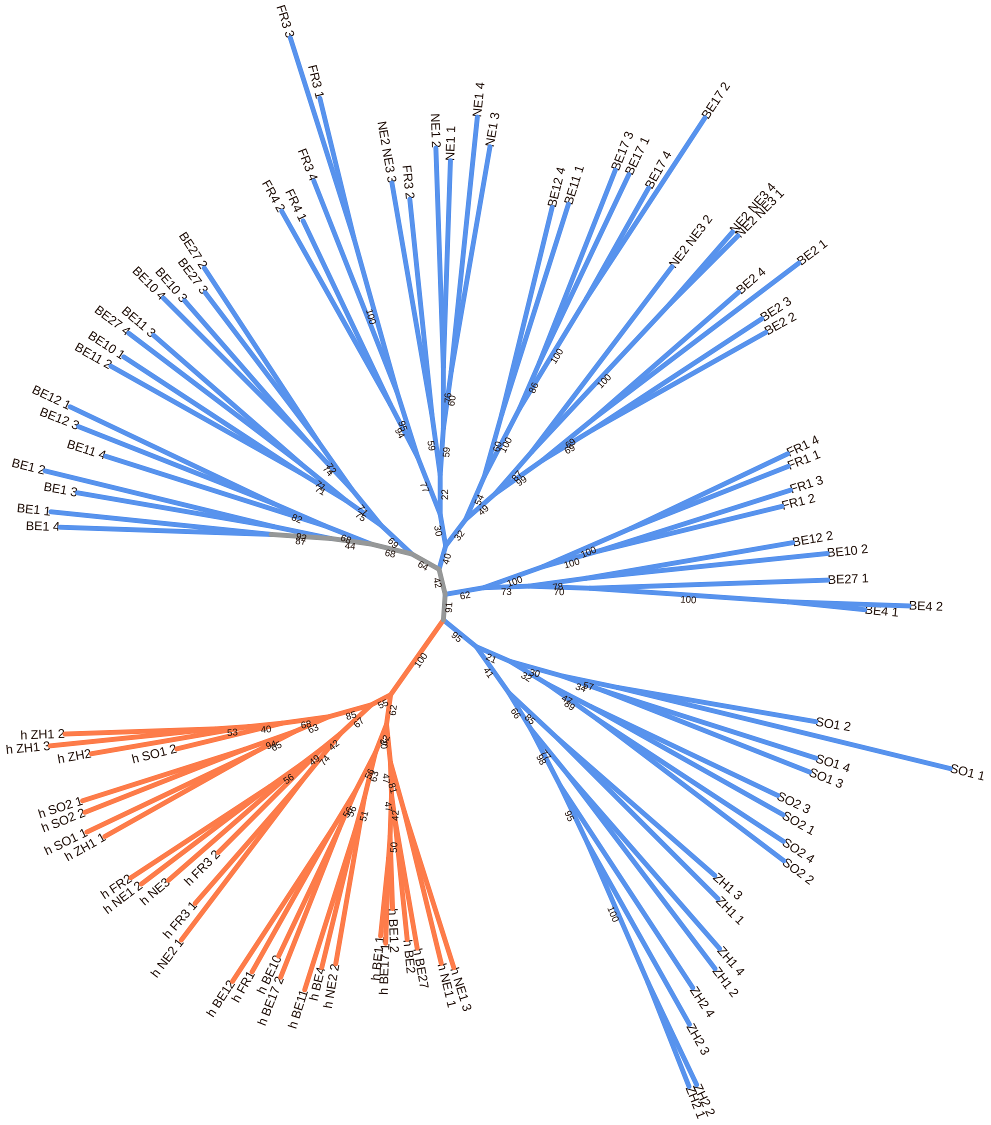

Figure S9. Maximum-likelihood phylogenetic tree based on 51,379 SNPs from the matched localities dataset, Related to Figure 3C. This tree is the same as in Figure 3C, but bootstrap support based on 10,000 resampling replicates is indicated for all branches, and individual sample identifiers (see Table S1) are given at the branch tips. Note the perfect bootstrap support for the reciprocal monophyly of the historical versus contemporary samples.

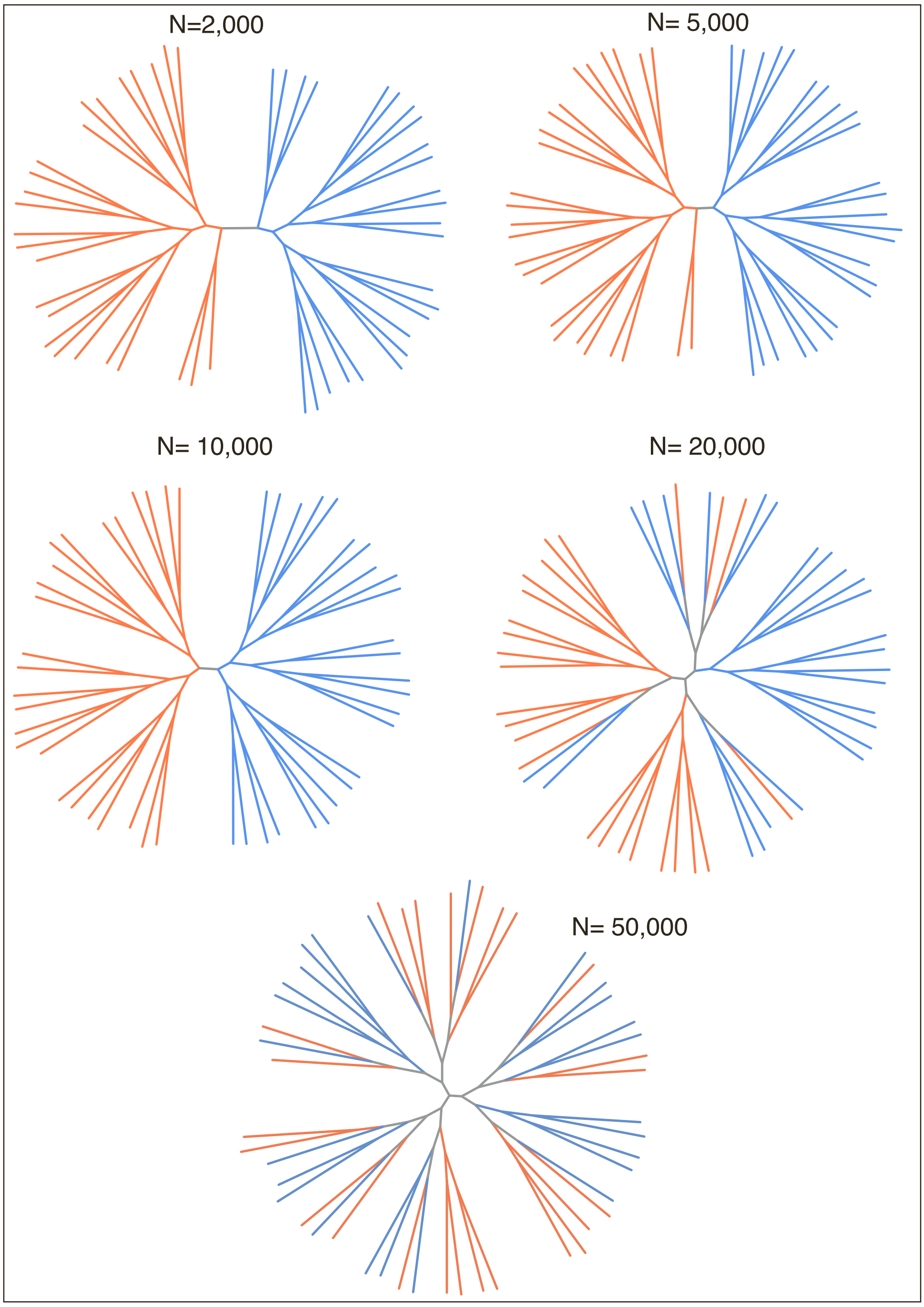

Figure S10. Phylogenetic trees for historical and contemporary samples simulated with evolution at different population sizes, Related to Figure 3C. This experiment started by generating 25 diploid individuals based on the allele frequencies observed empirically in the historical sample (50,601 SNPs). Allele frequencies were then allowed to evolve over 75 generations at different population sizes (range 2,000-50,000; indicated in the graphic), assuming drift as the only evolutionary process. Another 25 individuals were then derived from the allele frequencies resulting after evolution. Genotype data from all 50 individuals together served to construct maximum likelihood phylogenetic trees. Note the increasing phylogenetic structure between the two temporal groups with decreasing population size, resulting in reciprocal monophyly for very small population sizes. The figure presents the outcome of a single simulation per population size only, but replicates were performed and produced very similar results leading to identical conclusions.

Reference

1. Csillery, K., Francois, O. and Blum, M. G. B. (2012), abc: an R package for approximate Bayesian computation (ABC). Methods in Ecology and Evolution 3: 475479. doi:10.1111/j.2041-210X.2011.00179.x

2. Santiago, E., et al. (2020). Recent demographic history inferred by high-resolution analysis of linkage disequilibrium. Molecular Biology and Evolution, 37(12), 3642–3653. https://doi.org/10.1093/molbev/msaa169

3. Jónsson, H., Ginolhac, A., Schubert, M., Johnson, P. L. F., & Orlando, L. (2013). mapDamage2.0: Fast approximate Bayesian estimates of ancient DNA damage parameters. Bioinformatics, 29(13), 1682–1684. https://doi.org/10.1093/bioinformatics/btt193
